# Double agent: how *Escherichia coli* switches from commensal to pathogen in the urinary tract infection

**DOI:** 10.1007/s15010-026-02872-1

**Published:** 2026-07-03

**Authors:** Sabiha Gul

**Affiliations:** 1 University of Campania Luigi Vanvitelli ’ and AORN Ospedali dei Colli-Monaldi Hospital, Naples, Italy; 2 Institute for Biotechnology, Brandenburg University of Technology, Senftenberg, Germany

**Keywords:** *Uropathogenic Escherichia coli*, Phenotypic plasticity, Antibiotic tolerance, Urinary tract infection, Host-pathogen interaction

## Abstract

*Escherichia coli* exhibits a dual nature as both a beneficial gut commensal and the predominant cause of community-acquired urinary tract infections (UTIs) worldwide. This review synthesizes current evidence establishing phenotypic plasticity the capacity for dynamic, non-heritable, and reversible adaptation as a central determinant of uropathogenic *E. coli* pathogenesis, distinct from stable genetic resistance. From a multilayered perspective, a comprehensive analysis is provided of how genomic diversity, host-pathogen interactions at the bladder epithelium, and exposure to clinically relevant antibiotics collectively drive morphological and regulatory reprogramming. These adaptations include surface roughening, filamentation, and RpoS-mediated persistence, along with (p)ppGpp stringent response and EnvZ/OmpR two-component system signaling, which enhance bacterial survival independently of genetic resistance mutations. The review further discusses how these mechanisms establish a coordinated survival matrix, creating a fundamental disconnect between standard antibiotic susceptibility testing and the host-associated phenotypes that characterize actual infections. Unlike genetically resistant bacteria that grow at elevated antibiotic concentrations, phenotypically tolerant cells exhibit normal MICs but require prolonged killing times, explaining why recurrent UTIs occur despite appropriate therapy. Finally, recent advances, including phage vB_EcoP_P64441 combined with cefotaxime for biofilm disruption, glucose-mediated gentamicin tolerance targeting metabolic pathways, and HDAC inhibitors such as valproic acid for host-directed epigenetic reprogramming, offer new opportunities to break the debilitating cycle of recurrent UTIs affecting millions worldwide.

## Introduction

### Human intestinal microbiota and the role of *Escherichia coli*

The human gut microbiota includes *Escherichia coli* as a minor but consistent member, where it contributes to colonization resistance and immune tolerance [1]. However, when *E. coli* exits the intestinal tract and enters the urinary tract, it encounters a radically different environment that triggers adaptive changes [1, 2]. 

**Fig. 1:**
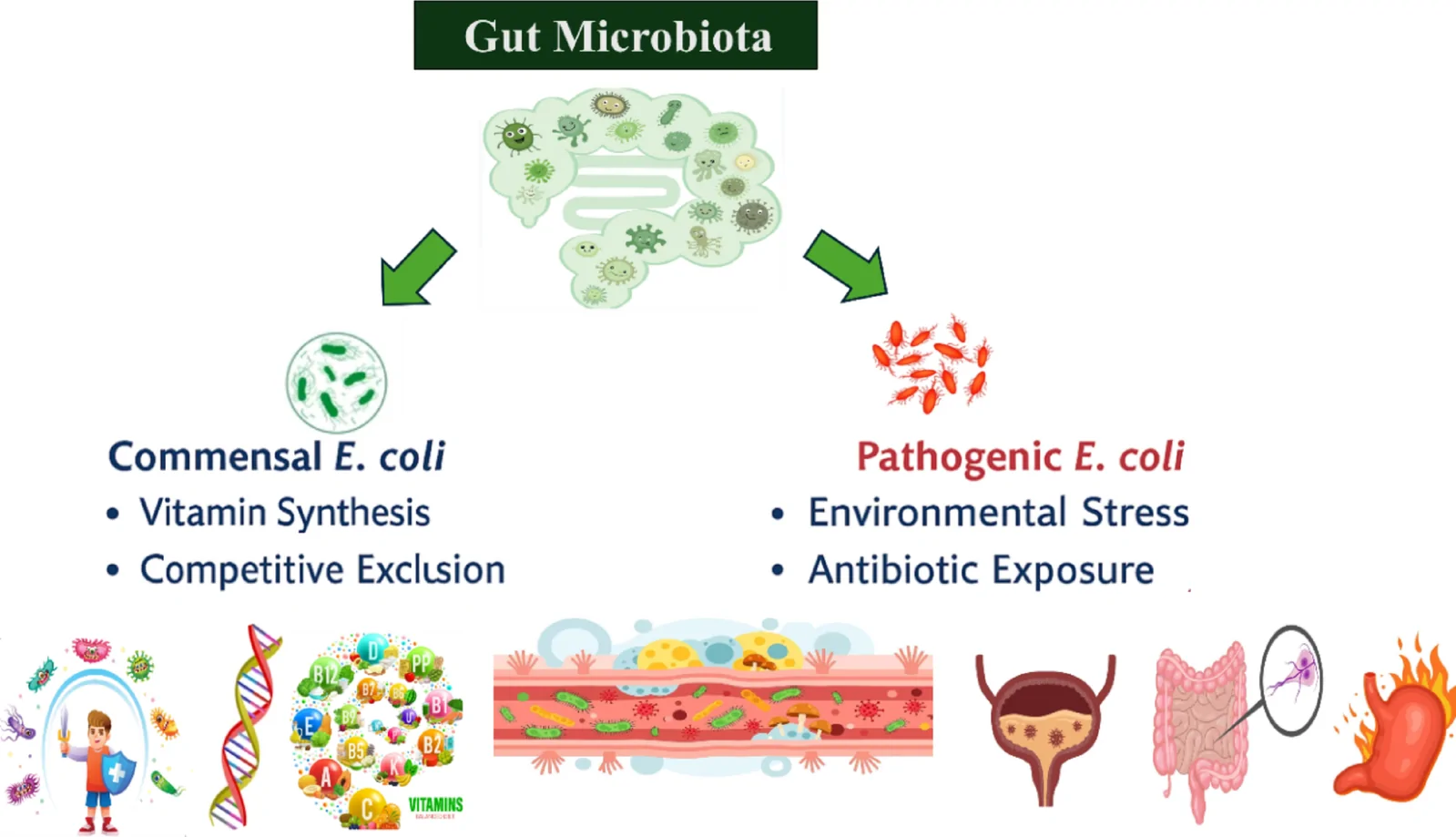
*Escherichia coli *in the gut microbiota.

The dual nature of *E. coli*, beneficial in the gut but potentially harmful in other niches, reflects its evolutionary success (Fig. 1). When introduced into extraintestinal environments such as the urinary tract or bloodstream, the bacterium can express specific adhesins, toxins, and iron-acquisition systems that facilitate colonization and survival. These adaptive processes form the basis of many clinically significant infections, particularly urinary tract infections (UTIs), which are among the most prevalent bacterial infections worldwide [3].

 This illustration shows how *E. coli* can function as both a beneficial commensal and a harmful pathogen within the intestinal environment. In balanced microbiota, commensal strains contribute to host health through vitamin synthesis and competitive exclusion of pathogens. Under environmental stress or antibiotic exposure, *E. coli* may acquire virulent traits, leading to inflammation, epithelial disruption, and disease symptoms. The diagram highlights the microbial plasticity of *E. coli* and its ability to shift roles depending on host and environmental conditions [2]

## Biological framework of *Escherichia coli*

### Taxonomic and genomic features

*E. coli* is a Gram-negative, facultatively anaerobic bacterium belonging to the Enterobacteriaceae family; its genomic plasticity enables rapid adaptation to environmental changes [4]. Several classification systems are used to distinguish *E. coli* strains, each with different relevance to pathogenic potential, as summarized in Table 1. The clinically relevant distinction, however, lies in pathogenic potential, discussed below [5–9].

**Table 1:** Overview of *E. coli* classification systems relevant to pathogenicity

Classification method	Basis	Relevance to phenotypic plasticity
Serotyping (O: H antigens)	Surface LPS and flagellar proteins	Primarily for strain identification; limited direct relevance
Phylogroup	Whole-genome or marker-based genotyping	Phylogroups B2 and D are enriched for UPEC virulence genes
MLST	Sequence variation in 7–8 housekeeping genes	Enables tracking of epidemic clones (e.g., ST131)

###  From commensal to pathogen: the pathotype concept

**Fig. 2:**
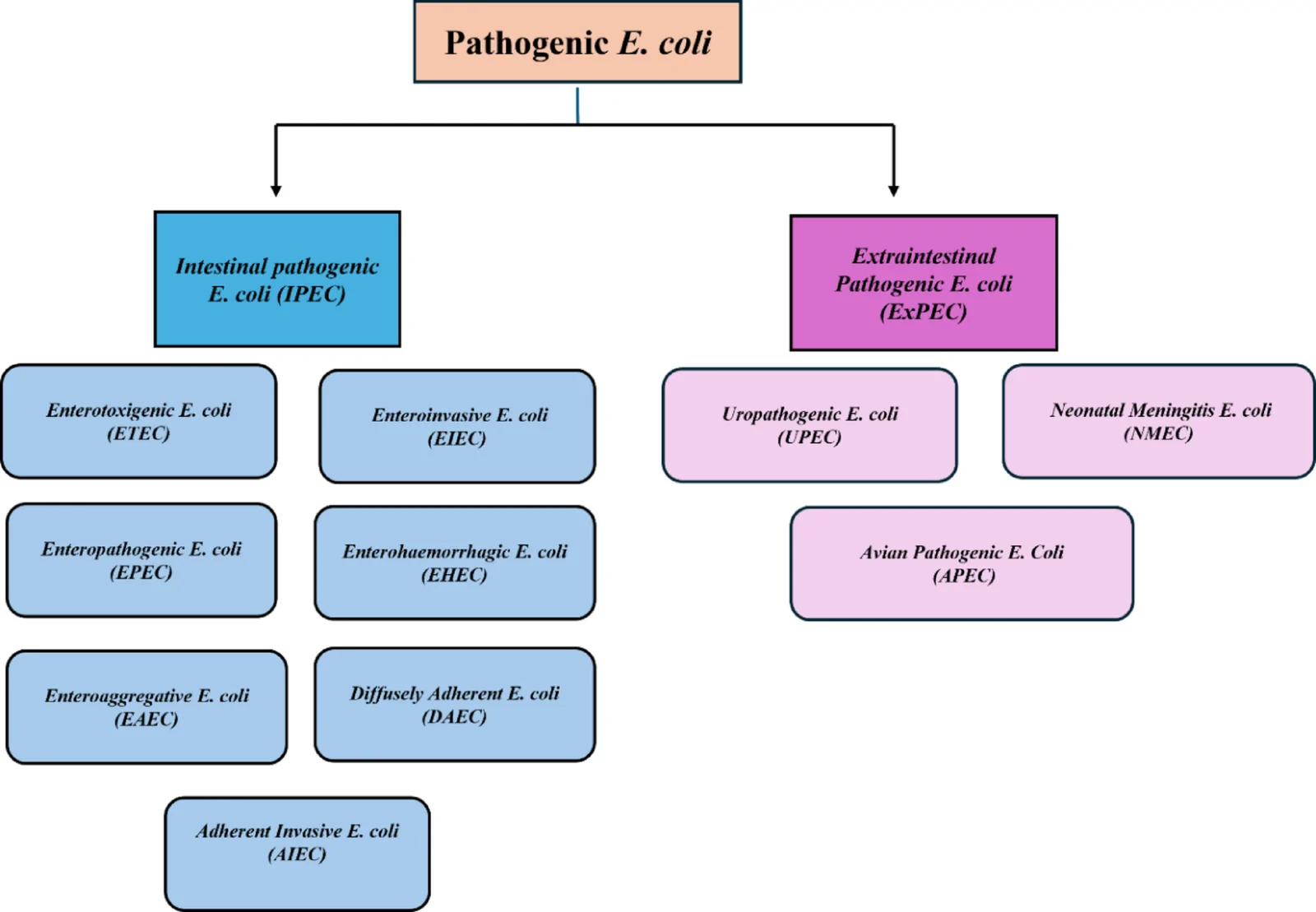
Classification of* E. coli* pathotypes.

Pathogenic *E.coli* strains are broadly divided into two major categories based on their site of infection and disease manifestation: intestinal pathogenic *E. coli (InPEC)* and extraintestinal pathogenic *E. coli (ExPEC)* (Fig. 2) [10].

Visual representation of major *Escherichia coli* pathotypes grouped by infection site. This schematic distinguishes intestinal (InPEC) and extraintestinal (ExPEC) variants, providing a clear overview of their clinical relevance and diversity [10]

*InPEC* strains are primarily associated with diarrheal diseases and target the gastrointestinal tract through distinct mechanisms. These include *enterotoxigenic E. coli (ETEC)*, which produces heat-labile and heat-stable enterotoxins that disrupt intestinal fluid balance; e*nteropathogenic E. coli (EPEC)*, which forms attaching and effacing lesions via a type III secretion system; *enteroaggregative E. coli (EAEC)*, known for its biofilm-like aggregative adherence and toxin production; and *enteroinvasive E. coli (EIEC)*, which invades colonic epithelial cells in a manner similar to Shigella, causing dysentery-like symptoms. Other recognized *InPEC* variants include *enterohemorrhagic E. coli (EHEC)*, which produces Shiga toxins and can lead to hemolytic uremic syndrome, and *diffusely adherent E. coli (DAEC)*, which exhibits a diffuse adherence pattern on epithelial cells [11].

In contrast, *ExPEC* strains can cause infections outside the intestinal tract and are often opportunistic pathogens. These include *uropthogenic E.coli (*UPEC*)*, the leading cause of urinary tract infections, which utilize adhesins like P fimbriae and type 1 fimbriae, hemolysins, and iron-scavenging siderophores to colonize and persist in the urinary tract. *Neonatal meningitis-associated E. coil (NMEC)* possesses a K1 capsule and invasion proteins that facilitate traversal of the blood-brain barrier, leading to meningitis in newborns. *Avian pathogenic E. coli (APEC)* causes systemic infections in poultry and shares many virulence traits with UPEC and *NMEC*, highlighting the cross-species adaptability of *ExPEC* strains [12].

The pathogenic potential of both *InPEC* and *ExPEC* strains is largely determined by their arsenal of virulence-associated genes (VAGs), which encode proteins involved in adhesion to host tissues, toxin production, nutrient acquisition (especially iron), and evasion of host immune responses. These genes are often located on mobile genetic elements such as plasmids, pathogenic islands, and bacteriophages, facilitating horizontal gene transfer between strains [13].

### Molecular signals and regulatory switches governing the commensal-to-pathogen transition

The transition from gut commensal to uropathogen is not a passive process but an active, signal-integrated decision. Four environmental signals encountered during ascension from the periurethral area to the bladder trigger this switch through specific two-component systems and transcription factors.

The sharp increase in osmolality encountered during the transition from the intestinal environment (~ 300 mOsm/kg) to human urine (typically 600–1200 mOsm/kg) acts as a potent environmental cue for UPEC [14]. Recent work shows that urine osmolarity rapidly remodels UPEC transcriptional programs and activates classical osmosensory pathways, including the EnvZ/OmpR two-component system, which responds to hyperosmotic stress [15, 16]. Contemporary regulatory studies further demonstrate that OmpR influences the fimS invertible element by modulating fimB/fimE recombinase activity, thereby biasing the fim operon toward the ON orientation under urinary tract conditions [17, 18]. This results in rapid induction of type 1 fimbriae, with urine exposure triggering fimbrial activation within minutes to the first hour of contact. Once expressed, the FimH adhesin mediates high-affinity binding to mannosylated receptors on bladder epithelial cells, enabling early colonization and invasion [19].

Iron limitation serves as a second major host-derived signal encountered by UPEC during the transition from the gut to the urinary tract. Human urine is profoundly iron-restricted, containing only trace levels of bioavailable Fe³⁺, which triggers rapid depression of the Fur regulon and induction of siderophore biosynthesis pathways [20]. Under these conditions, Fur inactivation permits strong upregulation of the iucABCD/iutA aerobactin operon, a high-affinity siderophore system that enables UPEC to extract iron from host transferrin and lactoferrin [21]. Recent transcriptomic studies demonstrate that aerobactin biosynthesis is activated within the first few hours of exposure to urine or bladder epithelial cells, and this response is essential for sustained bacterial growth and persistence in the iron-limited urinary tract environment [22, 23]. 

Leucine scarcity in urine provides an additional metabolic cue that modulates UPEC fimbrial expression. Whereas the gut lumen contains abundant branched-chain amino acids, human urine is markedly depleted in leucine and other essential amino acids. Recent metabolic profiling studies show that this nutrient limitation rapidly inactivates the global regulator Lrp, which requires leucine as a co-effector for DNA [24].Under leucine-starved conditions, Lrp repression of the fimA promoter is relieved, promoting increased transcription of type 1 fimbrial genes independently of the osmolality-driven EnvZ/OmpR pathway [25]. This metabolic mechanism acts in parallel with other urinary tract signals to ensure robust fimbrial expression during early bladder colonization.

Mechanical shear generated by pulsatile urine flow acts as a potent physical cue that promotes biofilm initiation in UPEC. Recent studies demonstrate that shear stress is sensed through envelope-associated mechano-transduction pathways, leading to activation of the biofilm master regulator CsgD, which controls curli fimbriae and cellulose biosynthesis [26, 27] Sustained exposure to moderate shear for 6–12 h induces a transcriptional shift from single-cell adhesion to multicellular, matrix-embedded aggregates, a phenotype strongly associated with recurrent and catheter-associated urinary tract infections [28]. This shear-responsive CsgD program enables UPEC to withstand urinary flow, persist on catheter surfaces, and establish biofilm-like communities that are highly tolerant to antibiotics and host defenses.

Although these host-derived cues operate on distinct timescales, they form an integrated regulatory cascade during early bladder colonization. Rapid signals such as osmolality shifts and amino-acid limitation modulate fimbrial regulators within the first hour of urine exposure, promoting early adhesion. Iron restriction drives siderophore induction over the subsequent few hours, supporting sustained growth in the nutrient-limited urinary tract. Under prolonged nutrient limitation and continuous shear stress conditions typical of catheterization biofilm-associated regulators such as CsgD become activated over longer periods, promoting the emergence of multicellular aggregates. This layered temporal logic helps explain why brief bladder exposure rarely results in infection, whereas indwelling catheters, which impose persistent shear and nutrient stress, strongly predispose to biofilm-associated, chronic UTI.

Importantly, the boundaries between pathotypes are not absolute. *E. coli* exhibits significant genomic plasticity, allowing commensal strains or those traditionally classified under one pathotype to acquire virulence traits characteristic of another. For example, non-UPEC strains may gain adhesins and siderophores that enable them to colonize the urinary tract under favorable conditions, such as immune suppression or antibiotic pressure. This phenomenon results in hybrid strains with mixed virulent profiles, complicating clinical diagnosis and treatment. It also underscores the evolutionary success and ecological versatility of *E. coli*, which can transition between harmless commensalism and pathogenicity depending on host and environmental factors [29].

## Host-pathogen interface in the urinary tract: the bladder environment and epithelial barriers

Urinary tract infections (UTIs) affect an estimated 150–200 million individuals globally each year, making them a major public health concern [30]. They disproportionately impact women due to anatomical and hormonal factors, as well as elderly individuals and patients with indwelling catheters, who are more susceptible to recurrent and complicated infections. The economic and clinical burden of UTIs includes frequent outpatient visits, hospitalizations, and rising antibiotic resistance, which complicates treatment and increases the risk of recurrence [31].

*E.coli* is the leading causative agent of UTIs, responsible for approximately 80–90% of community-acquired infections and nearly 50% of hospital-acquired (nosocomial) cases [32] Its dominance as a uropathogen stems from a sophisticated arsenal of virulence factors that enable it to colonize the urinary tract. These include adhesins such as type 1 fimbriae and P fimbriae, which mediate attachment to uroepithelial cells; toxins like hemolysin; and iron acquisition systems such as siderophores that help the bacterium survive in nutrient-limited environments. Importantly, *E. coli* can form intracellular bacterial communities (IBCs) within bladder epithelial cells, allowing it to evade immune responses and persist despite antibiotic treatment [33].

To study these interactions in vitro, researchers commonly use the 5637 human bladder epithelial cell line. 5637 is a cell line exhibiting epithelial morphology that was isolated from the urinary bladder of a 68-year-old, White male patient with grade II carcinoma [34], retains key features of native bladder epithelium, including its morphology and expression of surface receptors relevant to bacterial adhesion.5637 cells provide a reproducible and physiologically relevant model for investigating early infection events, such as bacterial attachment, invasion, and intracellular survival. When exposed to UPEC, these cells exhibit patterns that closely mimic the initial stages of infection observed in vivo [35].

One of the most informative aspects of this model is the ability to observe and classify *E. coli* adhesion phenotypes. These patterns clumpy, chain like, microcolony, and diffuse reflect distinct bacterial strategies for colonization and immune evasion. Clumpy and microcolony phenotypes are often associated with biofilm-like behavior, characterized by dense bacterial aggregates that enhance survival under stress conditions, including antibiotic exposure. These structures may serve as reservoirs for persistent infection and contribute to treatment failure. In contrast, chain-like and diffuse adhesion patterns suggest more dispersed colonization, often linked to rapid bacterial proliferation and reduced aggregation. These phenotypes may be more vulnerable to host defenses but can facilitate swift expansion within the urinary tract [36].

The dynamic nature of these adhesion phenotypes is influenced by both bacterial genotype and environmental conditions, such as nutrient availability, host cell signaling, and antibiotic pressure. Understanding how these phenotypes correlate with virulence traits, antibiotic susceptibility, and persistence is critical for developing targeted therapies and improving diagnostic precision [37]. This study focuses on characterizing these adhesion behaviors in detail, aiming to uncover their role in UTI pathogenesis and their potential as biomarkers for predicting clinical outcomes.

## Adhesion phenotypes on bladder epithelium: molecular basis of host-cell attachment

Adhesion to the bladder epithelium is a critical early step in urinary tract colonization, governed by sophisticated molecular interactions extensively characterized using the human bladder epithelial cell line 5637. Research has progressively revealed the complexity of this process, evolving from identification of essential bacterial adhesins to discovery of specific host receptors, phenotypic diversity, environmental modulation, and most recently, host epigenetic reprogramming summarized in Table 2. Foundational studies established the indispensability of type 1 pili and FimH adhesin, while subsequent work identified the specific host receptor α3β1 integrin and downstream FAK signaling. Phenotypic analysis later classified four distinct adhesion patterns, with clump formation specifically observed in the 5637 model [38]. Host environmental factors, including immune components and physical forces, were shown to modulate these interactions. Most recently, the field has expanded to include bacterial regulatory mechanisms and host epigenetic responses that create permissive landscapes for bacterial persistence and recurrence. This progression demonstrates an evolving understanding from simple receptor-ligand binding to a complex interplay of bacterial phenotyping, physical forces, and deep host-cell reprogramming.

**Table 2:** Key findings on adhesion phenotypes in bladder epithelium

References	Key finding	Model system	Mechanism
[39]	Type 1 pili indispensable for UPEC invasion; FimH essential	5637 bladder epithelial cells	Bacterial adhesion via FimH on type 1 pili
[40]	Identified α3β1 integrin as host receptor; FimH-integrin activates FAK signaling	5637 bladder epithelial cells	Receptor-ligand interaction; FAK signaling drives internalization
[41]	Surfactant protein D (SP-D) inhibits bacterial adhesion	Bladder epithelial cells	Host immune components modulate adhesion
[36].	Classified four adhesion phenotypes: diffuse, microcolony, chain, clump	5637 bladder epithelial cells	Phenotypic diversity; clump formation specific to 5637
[42]	Physiological shear stress reduces FimH-mediated attachment	Bladder epithelial cells	Physical forces influence adhesion
[43]	UPEC induces lasting epigenetic reprogramming in urothelial cells	Urothelial cells	Epigenetic alterations affect host defenses
[38]	Confirmed clumpy adhesion phenotype	5637 bladder epithelial cells	Validates earlier phenotypic observations
[44]	c-di-AMP enhances invasion via flagellin elongation	Related pathotypes	Bacterial signaling regulates adhesion/invasion
[45]	UPEC induces epigenetic reprogramming promoting bacterial persistence	Urothelial cells	Creates permissive landscape for recurrence

## Phenotypic plasticity as a survival strategy

*E. coli* exhibits substantial phenotypic plasticity, allowing it to dynamically adjust to changing environmental and host-associated conditions. This adaptability is crucial for its survival and pathogenic success, especially during UTIs, where the bacterium encounters a range of stressors. These include nutrient limitation, osmotic fluctuations, oxidative stress, antimicrobial exposure, and immune system pressures. In response, *E. coli* can reprogram its gene expression, alter its surface structures (such as fimbriae and outer membrane proteins), and shift its metabolic pathways to optimize survival [46].

These physiological changes directly influence key behaviors such as motility, adhesion, and biofilm formation. For instance, under nutrient-deprived or antibiotic-stressed conditions, *E. coli* often transitions into a sessile lifestyle, forming aggregates or biofilms that provide mechanical protection and enhance persistence. Biofilm-like structures are particularly important in chronic infections, as they shield bacteria from immune clearance and reduce antibiotic penetration [10].

**Fig. 3:**
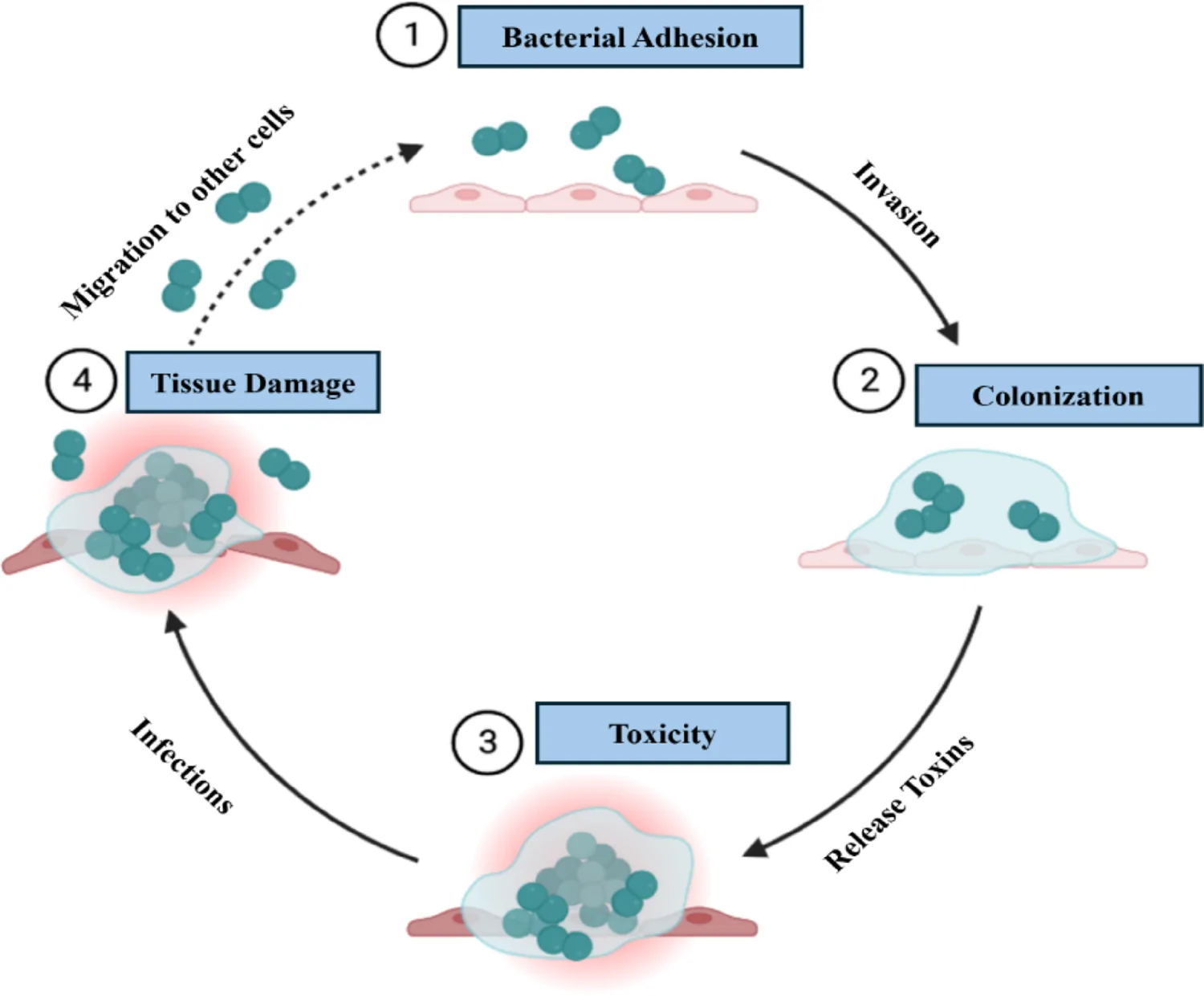
Stages of *Escherichia coli *infection and pathogenesis**.**

Phenotypic heterogeneity also contributes to antibiotic tolerance, a reversible, non-genetic state where bacteria survive antimicrobial treatment without acquiring resistance mutations. Sessile or aggregated cells typically show reduced metabolic activity and activate stress-response regulons, making them less susceptible to antibiotics. Over time, these adaptive traits can serve as a steppingstone toward stable antimicrobial resistance, especially in recurrent or poorly treated infections [47]. The infection process itself is shaped by these adaptive transitions. As illustrated in (Fig.3)

Infection cycle of *E. coli* (UPEC), showing bacterial adhesion, colonization, toxin release, and tissue damage. These stages reflect the dynamic interaction between UPEC and host tissues during urinary tract infection [48].

*E. coli* progresses through distinct stages: adhesion to host cells, colonization, toxin release, and tissue damage, often followed by invasion and migration to adjacent cells. Each stage reflects a shift in bacterial phenotype tailored to the local microenvironment. This cyclical progression underscores the importance of phenotypic plasticity in sustaining infection and evading host defenses [49].

## Antibiotic-induced morphological adaptation

### Distinguishing phenotypic tolerance from genetic resistance: experimental evidence

A critical conceptual distinction must be made before examining morphological changes. Clinical failure of antibiotic therapy can result from two fundamentally different bacterial states: genetic resistance and phenotypic tolerance. These are distinguished by four experimentally measurable parameters.

#### Parameter 1: heritability

Genetic resistance is heritable, whereas phenotypic tolerance is not. A genetically resistant bacterium produces progeny that remains resistant even when cultured in antibiotic-free media. In contrast, phenotypically tolerant cells revert to full susceptibility once the antibiotic stress is removed [50]. Supporting evidence comes from Uzoechi and Abu-Lail [51, 52], who showed that ampicillin-exposed *E. coli* developed increased surface roughness, stiffness, and adhesion features of a tolerant state but returned to baseline morphology and susceptibility after overnight growth in antibiotic-free Mueller–Hinton broth. Conversely, a blaTEM-positive resistant control strain maintained stable resistance through more than 20 passages without antibiotic selection, demonstrating the heritable nature of genetic resistance.

#### Parameter 2: minimum inhibitory concentration (MIC)

Genetic resistance and phenotypic tolerance differ fundamentally in their effects on the minimum inhibitory concentration (MIC). Genetic resistance elevates the MIC resistant *E. coli* may exhibit ampicillin MIC values of 32 µg/mL compared with 4 µg/mL in susceptible strains whereas phenotypic tolerance leaves the MIC unchanged [53]. Windels et al.[54]. demonstrated this distinction in a cohort of 50 recurrent-UTI isolates: tolerant strains, defined by survival at 4× MIC ampicillin for 6 h, displayed MICs of only 2–8 µg/mL, identical to fully susceptible controls. Despite normal MICs, these tolerant isolates required markedly prolonged exposure 6.2 ± 1.8 h to achieve 99.9% killing, compared with just 1.8 ± 0.4 h for non-tolerant isolates (*p* < 0.001). This confirms that tolerance prolongs killing time without altering MIC, distinguishing it sharply from heritable genetic resistance.

#### Parameter 3: minimum duration for killing (MDK)

The MDK₉₉ the time required to kill 99% of a bacterial population is the most direct quantitative measure of antibiotic tolerance. Genetically resistant bacteria do not exhibit meaningful killing at susceptible drug concentrations, whereas tolerant bacteria are ultimately killed but require prolonged exposure despite having normal MICs. Pérez-Villalobos et al. (2025) [55] demonstrated this distinction using ciprofloxacin-treated adherent-invasive *E. coli* (AIEC) clinical isolates: high-persistence strains exhibited an MDK₉₉ of 8.4 h, compared with 2.1 h for low-persistence controls, even though both groups shared an identical MIC of 0.25 µg/mL. Crucially, MDK₉₉ not MIC predicted treatment outcomes in a mouse model of recurrent infection, underscoring that tolerance manifests as delayed killing rather than reduced susceptibility.

#### Parameter 4: genetic basis

Genetic resistance arises from stable DNA alterations such as point mutations in *gyrA* (S83L) or *parC* (S80I), or acquisition of resistance genes like *bla*CTX-M-15 that permanently elevate MICs and confer heritable survival advantages. In contrast, phenotypic tolerance requires no genetic change; it is driven by reversible physiological and epigenetic adaptations including RpoS activation, (p)ppGpp accumulation, and toxin–antitoxin system engagement. Abdelwahed et al. [56] demonstrated this clearly: transcriptomic profiling of *E. coli* exposed to multiple antibiotics revealed upregulation of 147 stress-response genes (including *rpoS*, *dnaK*, *ibpA*, *clpB*) and downregulation of 89 genes involved in translation (*rpoB*, *rpsL*, *fusA*). Crucially, whole-genome sequencing of tolerant populations detected no SNPs in known resistance loci, confirming that tolerance reflects a non-genetic, regulatory state rather than heritable resistance.Table 3.

**Table 3:** Distinguishing features of genetic resistance vs. phenotypic tolerance in E. coli

Parameter	Genetic Resistance	Phenotypic Tolerance
Heritability	Yes (stable across generations)	No (reverts without drug pressure)
MIC (minimum inhibitory concentration)	Elevated (above clinical breakpoint)	Normal (within susceptible range)
MDK₉₉ (time to kill 99% of bacteria)	Not applicable (grows in drug)	Prolonged (3-8x longer than susceptible)
Genetic change required	Mutation or acquired resistance gene	None (transcriptional/epigenetic only)
Clinical consequence	Treatment failure from day 1 of therapy	Late relapse after therapy ends
Standard AST detection	Yes (disk diffusion, Etest, broth microdilution)	No (requires time-kill or persister assays)
Reversibility without drug pressure	No (stable)	Yes (reverts to susceptible phenotype)

In summary, these comparative parameters clearly delineate genetic resistance from phenotypic tolerance. Resistance is a heritable, mutation-driven state that elevates MICs, is readily detected by standard AST methods, and causes immediate treatment failure. In contrast, tolerance is a reversible, non-genetic survival strategy characterized by normal MICs but markedly prolonged killing times, escaping routine diagnostics and predisposing patients to relapse after therapy ends. Together, these distinctions underscore why tolerance must be evaluated alongside resistance to fully understand antibiotic treatment outcomes. The next step is to examine the molecular systems that generate this tolerant state. Phenotypic plasticity does not arise randomly; it is orchestrated by specific regulatory pathways that sense antibiotic stress and reprogram cellular physiology. The following section outlines the major regulatory networks RpoS, (p)ppGpp, and key two-component systems that drive antibiotic-induced adaptation in *E. coli*.

### Regulatory pathways governing phenotypic plasticity

Phenotypic plasticity during antibiotic exposure is not a passive or stochastic process. Instead, *E. coli* activates defined regulatory networks that sense environmental stress, remodel cellular physiology, and generate the tolerant morphological states described later in this section. Three major pathways RpoS, the (p)ppGpp stringent response, and envelope-stress two-component systems have been experimentally validated as central drivers of antibiotic-induced adaptation [57].

#### The RpoS (σ⁽ˢ⁾) regulon: master controller of stress-induced plasticity

RpoS is the principal general-stress sigma factor that redirects RNA polymerase toward genes required for survival during nutrient limitation, oxidative and osmotic stress, and antibiotic exposure. During exponential growth, RpoS is rapidly degraded by the ClpXP protease, but antibiotic stress stabilizes RpoS, enabling activation of more than 500 protective genes [58]. Key RpoS-regulated loci relevant to morphological adaptation include *katE* (catalase protecting against oxidative bursts), *otsAB* (trehalose biosynthesis for osmoprotection), *csgBA* (curli fimbriae contributing to biofilm matrix formation), and *bolA* (a morphology regulator promoting rounding and filamentation).Valencia et al. (2024) [59] shows that high-RpoS UPEC isolates exhibit a 5.7-fold increase in survival after 6 h of ampicillin exposure compared with low-RpoS isolates, despite identical MICs; deletion of *rpoS* abolished this advantage, confirming RpoS as a causal determinant of antibiotic-induced tolerance.

#### The (p)ppGpp stringent response: metabolic reprogramming and growth slowdown

The alarmone nucleotides ppGpp and pppGpp collectively (p)ppGpp accumulate when ribosomes stall during amino acid starvation, oxidative stress, or antibiotic-induced translational damage, triggering the stringent response [60] By binding RNA polymerase, (p)ppGpp globally reprograms transcription, repressing rRNA and tRNA synthesis, slowing DNA replication and growth, and activating amino acid biosynthesis and stress-response pathways. During bladder epithelial cell infection, internalized UPEC rapidly encounters amino acid limitation, leading to (p)ppGpp accumulation and a shift from ~ 45 min to > 180 min doubling time [61]. These slow-growing, (p)ppGpp-high cells exhibit up to 100-fold increased survival against aminoglycosides, which preferentially kill rapidly dividing bacteria. Importantly, (p)ppGpp synergizes with RpoS by inhibiting ClpXP-mediated RpoS degradation, creating a positive feedback loop that stabilizes the tolerant state [62].

#### Two-component systems (EnvZ/OmpR and CpxAR): envelope remodeling under stress

Two-component systems play a central role in sensing environmental stress and remodeling the bacterial envelope to modulate antibiotic influx. The osmosensory EnvZ/OmpR system is activated by the high osmolality of urine (600–1200 mOsm/kg), leading to EnvZ-mediated phosphorylation of OmpR, which upregulates *ompC* and represses *ompF* [18].This porin shift reduces the entry of hydrophilic antibiotics particularly β-lactams and tetracyclines by 60–80%, yet does not alter MICs measured in low-osmolality AST media that fail to activate EnvZ/OmpR [63]. In parallel, the CpxAR system responds to misfolded periplasmic proteins, lipoprotein defects, and β-lactam-induced cell wall stress Activated CpxR induces *degP*, *dsbA*, and *cpxP*, enhancing periplasmic quality control and envelope repair. Strains with constitutively active CpxAR exhibit up to 10-fold increased survival during ampicillin exposure, reflecting strengthened envelope maintenance and reduced lysis [64, 65].

### Clinical correlation between pathway activity and recurrence risk

Clinical evidence increasingly shows that phenotypic plasticity rather than classical antibiotic resistance underlies recurrence in a substantial subset of UTI patients. Foundational work by Mysorekar and Hultgren (2006) [66] demonstrated that quiescent intracellular reservoirs formed during acute infection enable long-term persistence and are a major driver of recurrence, even when isolates remain fully susceptible on AST. Subsequent reviews, including Engelhardt et al. (2025) [67], highlight that activation of stress-response pathways such as RpoS and the (p)ppGpp stringent response enhances intracellular survival, promotes biofilm formation, and facilitates the establishment of these reservoirs. Together, these clinical and mechanistic insights indicate that recurrence is frequently driven by stress-induced persistence programs rather than heritable resistance determinants, explaining why many recurrent UTI cases occur despite apparently appropriate antibiotic therapy.

## Morphological and physiological responses to antibiotic exposure

Exposure of *E. coli* to antibiotics, whether at sub-inhibitory or lethal concentrations, does not necessarily result in immediate bacterial death. Instead, antibiotic stress frequently triggers profound morphological and physiological adaptations. Under such conditions, *E. coli* exhibits characteristic phenotypic changes including filamentation, cell-size reduction, and increased surface roughness that collectively enhance adhesion and promote tolerance independent of genetic resistance mutations [68]. These adaptive responses arise through multiple mechanisms summarized in Table 4. Antibiotic exposure can directly modify cell-surface architecture, increase adhesiveness and facilitate population persistence. In parallel, global transcriptomic reprogramming occurs, with up-regulation of biofilm-associated and stress-survival genes and down-regulation of energy-intensive metabolic pathways. Recent evidence highlights the stress-response sigma factor RpoS as a central regulator of these tolerance phenotypes, with high-persistence morphotypes documented in clinically relevant strains following antibiotic exposure. Collectively, these findings demonstrate that antibiotic stress acts as a potent modulator of bacterial phenotype, driving survival through dynamic morphological and regulatory adaptations rather than through stable genetic resistance.

**Table 4:** Experimental evidence for phenotypic plasticity in antibiotic-exposed *E. coli*

Strains	Stress condition	Phenotypical change	Survival/tolerance outcome	References
A5/A9	Ampicillin	Increased cell elasticity and surface roughness	Enhanced survival	[51, 52]
A5/A9 (MDR)	Ampicillin	Reduced cell size; increased adhesion	Persisted populations	[52]
K-12 MG1655	Antibiotic/oxidative/thermal stress	Global transcriptomic reprogramming	Biofilm and stress adaptation	[56]
Clinical isolates	Multiple antibiotics	RpoS-regulated stress response	Tolerance and persistence	[59]
AIEC	Ciprofloxacin	High-persistence phenotype	Recurrence risk increased	[55]

## Physical stress and cell wall remodeling

Phenotypic plasticity in bacteria extends far beyond chemical challenges, encompassing adaptive responses to direct physical stress. As mentioned in (Table 5), physical disruption from nanostructured surfaces triggers profound morphological reprogramming in *E. coli*, including increased sphericity, expanded periplasmic density, and cell wall thickening alterations so significant that Gram-negative cells begin to stain like Gram-positive organisms [69] These structural changes carry functional consequences, directly altering antibiotic susceptibility profiles and demonstrating a mechanistic link between mechanical stress, cell wall architecture, and drug tolerance. Beyond this specific example, phenotypic plasticity represents a broader non-genetic survival strategy enabling bacterial adaptation to hostile environments. Recent work has begun unraveling the genetic architecture underlying such responses, identifying specific regulatory genes governing these adaptive shifts in other species [70, 71], while mathematical models illustrate the population-level trade-offs of phenotype switching, showing how transitioning to slow-growing, tolerant states can optimize survival under stress [72].

**Table 5:** Key studies on physical stress-induced phenotypic plasticity in bacteria

Focus area	Bacterial species/model	Key finding	Phenotypical change	References
Physical stress response	*E. coli*	ZnO nanorod collisions induce morphological reprogramming; Gram-negative cells stain like Gram-positive	↑ Sphericity ↑ Periplasmic density (15%) ↑ Cell wall thickness (30%)	[69]
Genetic regulation	*Staphylococcus aureus*	Identified genes governing plastic responses	Regulatory shifts enabling adaptation	[70]
Population dynamics	Bacterial populations (mathematical model)	Phenotype switching trade-offs modeled; slow-growing states optimize survival	Transition to tolerant phenotype under stress	[72]

## Planktonic vs. host-associated states

Bacterial susceptibility differs drastically between free-floating (planktonic) and surface-associated states. Host-cell adhesion or biofilm formation creates protective niches that enhance antibiotic tolerance [73]. Standard planktonic AST often underestimate resistance relevant to infections. Transcriptomic comparisons confirm that host-cell association activates distinct survival pathways. For instance, *Shiga toxin-producing E. coli* (*STEC*) undergoes large-scale transcriptional reprogramming during epithelial infection, optimizing metabolism for intracellular survival [74]. Clinically, showed that UPEC strains from recurrent UTIs (hiding their identity) display altered virulence and adhesion gene expression in host-associated states [75]. Correlated antibiotic resistance, biofilm formation, and virulence determinants in UPEC from Egyptian hospitals, further connecting clinical survival with adhesion capacity.

## Methodological caveats: bridging the gap between laboratory models and clinical reality

### Limitations of the 5637 bladder epithelial cell model

The 5637-bladder epithelial cell line has been widely used to study UPEC adhesion and invasion, and this review has cited several key studies using this model [36, 38]. However, it is important to recognize that 5637 cells are not normal urothelial cells. This cell line was isolated from a 68-year-old male patient with grade II transitional cell carcinoma of the urinary bladder [34]. Cancer cells differ from normal human urothelial cells (NHUC) in three ways that may influence bacterial adhesion studies.

### Limitation 1: altered surface glycosylation

Malignant transformation changes the glycoproteins and glycolipids expressed on the cell surface. Specifically, bladder cancer cells overexpress Tn antigen (GalNAcα1-O-Ser/Thr) and sialyl-Tn antigen, which are rare on normal urothelium. Some bacterial adhesins, including UPEC FimH, recognize specific glycan structures. The affinity of FimH for 5637 cells may therefore differ from its affinity for normal bladder epithelium [77]. 

### Limitation 2: modified integrin expression

The FimH receptor α3β1 integrin is upregulated in many bladder cancer cell lines, including 5637, compared to normal urothelial cells. This upregulation may influence UPEC invasion rates observed in vitro. Studies comparing 5637 to primary NHUC have found that UPEC invasion efficiency is 2–3 fold higher in 5637 cells, suggesting that the carcinoma-derived line may provide a different context for intracellular bacterial load assessment [40]. 

### Limitation 3: altered cytokine secretion

5637 cells produce lower baseline levels of pro-inflammatory cytokines (IL-6, IL-8) compared to NHUC. This means that host immune responses measured in 5637 infection models may underestimate the true inflammatory response that occurs in patients. Studies using NHUC show significantly higher IL-8 secretion upon UPEC infection compared to 5637 cells [43]. 

### Recommendation for readers and future studies

Findings from 5637 bladder-cell infection models including the clumpy adhesion phenotype described by Khan et al. (2024) [38] represent an important contribution to understanding host-associated phenotypic plasticity in *E. coli*. To strengthen and generalize these observations, future studies should validate them across additional model systems with increasing physiological relevance. The T24 bladder carcinoma line offers a less transformed alternative to 5637 while remaining commercially accessible [78]. Primary normal human urothelial cells (NHUC) provide a non-transformed, karyotypically normal model that more closely reflects native bladder epithelium, though they introduce higher cost and donor-to-donor variability [79]. At the highest level of biological fidelity, bladder organoids recreate three-dimensional architecture and cell–cell junctions, enabling investigation of adhesion and persistence within a structured urothelial environment, although standardization across laboratories remains limited. Together, these complementary systems offer a logical progression for validating phenotypes observed in 5637 cells and for advancing mechanistic insight into antibiotic-induced adaptation and persistence.

### The phenotypic tolerance gap: why standard AST misrepresents in vivo drug activity

Current antibiotic susceptibility testing (AST) performed under CLSI or EUCAST guidelines is optimized for standardization, not physiological relevance. As a result, laboratory MIC values often fail to predict clinical outcomes in urinary tract infections (UTIs). This mismatch termed the phenotypic tolerance gap arises because AST conditions differ fundamentally from the in vivo environment of the urinary tract [80]. 

#### Four key differences between standard AST and in vivo infection

*First*, standard AST evaluates planktonic, exponentially growing bacteria. In contrast, during UTI, *uropathogenic E. coli* occupy multiple phenotypic states, including urothelial-adherent cells, intracellular bacterial communities (IBCs), quiescent reservoirs, and slow-growing persisters [81, 82]. Each state exhibits distinct antibiotic susceptibility profiles that are not captured by planktonic MIC testing.

*Second*, AST uses nutrient-rich Mueller–Hinton broth, whereas the urinary tract contains nutrient-poor urine with high osmolality (600–1200 mOsm/kg) and variable pH (5.5–7.5). These conditions activate stress-response pathways ncluding RpoS and the (p)ppGpp stringent response that increase antibiotic tolerance without altering MIC [83, 84].

*Third*, AST is performed under static conditions. In vivo, the bladder experiences pulsatile urine flow generating shear stress of 0.5–5 dyn/cm². Shear forces alter bacterial gene expression, including downregulation of FimH-mediated adhesion and modulation of biofilm pathways demonstrated that physiological shear stress reduces FimH-dependent attachment by 60–80% compared with static conditions [42]. 

*Fourth*, AST lacks host immune components. In vivo, bacteria encounter neutrophils, complement, antimicrobial peptides, and antibodies, all of which shape antibiotic efficacy.Some antibiotics synergize with innate immunity, while others rely almost entirely on host defenses interactions that cannot be reproduced in standard AST [85, 86]. 

## Conclusions and future directions

The evidence synthesized in this review establishes phenotypic plasticity as a central determinant of *Escherichia coli* pathogenesis in urinary tract infections. From its dual existence as a commensal gut inhabitant and an opportunistic pathogen, to its display of distinct adhesion morphotypes on bladder epithelium, *E. coli* demonstrates a remarkable capacity for adaptive change [36, 38]. Critically, exposure to antibiotic stress triggers morphological and regulatory shifts including surface roughening, cell-size reduction, filamentation, and RpoS-mediated persistence programs that enhance bacterial survival without requiring stable genetic resistance. These plastic responses contribute directly to the cycle of recurrent infection that poses such significant clinical challenges.

## Summary of key findings

This review has synthesized current knowledge across several interconnected domains. First, we examined the biological framework of *E. coli*, highlighting its genetic diversity through serotyping, phylogroups, and MLST classification, which provides the underlying basis for phenotypic variation. Second, we explored the pathotype concept, demonstrating how genomic plasticity enables *E. coli* to transition between commensal and pathogenic states through acquisition of virulence-associated genes via mobile genetic elements. Third, we analyzed the host-pathogen interface in the urinary tract, focusing on the 5637-bladder epithelial cell model and the four distinct adhesion phenotypes—clumpy, chain-like, microcolony, and diffuse that reflect different bacterial strategies for colonization and immune evasion. Fourth, and most critically, we examined how antibiotic and physical stressors drive phenotypic plasticity, with studies demonstrating that ampicillin exposure induces surface roughening and increased adhesiveness [51, 52] that transcriptomic reprogramming activates biofilm-associated genes [56], and that regulatory networks involving RpoS govern persistence phenotypes [55, 59]. Together, these findings reveal that *E. coli* survival under stress is not merely a function of pre-existing genetic traits but involves dynamic, reversible adaptations that enhance fitness in hostile environments [72]. 

## Clinical implications

The recognition that phenotypic plasticity drives UPEC pathogenesis carries profound clinical implications. Current antibiotic susceptibility testing (AST) relies on planktonic bacteria under standard laboratory conditions, which fails to capture the host-associated and stress-induced phenotypes that characterize actual infections [87]. Bacteria that appear susceptible in standard assays may exhibit tolerance when attached to bladder epithelium or when forming intracellular bacterial communities. These disconnects between laboratory testing, and in vivo behavior helps explain why apparently susceptible strains cause recurrent infections despite appropriate antibiotic therapy.

Moreover, the phenomenon of antibiotic-induced phenotypic changes suggests that treatment itself may inadvertently promote bacterial persistence. Sub-inhibitory antibiotic concentrations which may occur at infection sites due to poor penetration, incomplete dosing, or pharmacokinetic variability can trigger the very adaptive responses that enable survival. This creates a concerning paradox: antibiotics designed to eliminate infection may, under certain conditions, promote the formation of persister populations that seed recurrence. The observation that different antibiotics may induce distinct phenotypic changes (e.g., filamentation versus cell-size reduction) raises the possibility that antibiotic choice could influence not only immediate bacterial killing but also the likelihood of subsequent recurrence [76]. 

## Emerging therapeutic strategies targeting phenotypic plasticity

Recent studies have identified several promising strategies targeting UPEC phenotypic plasticity, as summarized in Table 6.

**Table 6:** Emerging combination strategies for targeting phenotypic plasticity in UPEC Infections

Clinical scenario	Proposed strategy	Key finding	References
Biofilm-associated UTI	Phage vB_EcoP_P64441 + cefotaxime	Phage combined with antibiotic inhibits UPEC biofilm formation on catheters	[88]
UTI in diabetic patients	Consider glucose effect on gentamicin	Glucose (2 mg/mL) reduces gentamicin efficacy via rpoS mutations	[89]
Persistent UTI	Target filamentation mechanism	Filamentation helps UPEC avoid macrophage engulfment; occurs independently of sulA/ymfM	[90]
Chronic/recurrent UTI	Phage therapy, QS inhibitors, or nanoparticles	Emerging therapies targeting UPEC biofilms show promise	[91]
Personalized treatment	Whole genome sequencing	Genomic plasticity means virulence factors vary by strain	[92]

### Phage therapy for UPEC biofilm control

Li et al. (2025) [88] isolated and characterized a novel UPEC phage, vB_EcoP_P64441, belonging to the family Autographiviridae. The phage demonstrated stability at temperatures from 4 to 50 °C and pH range of 3–11, with a latent period of 4 min and burst size of 10 PFU/cell. Whole genome sequencing revealed a 42,960 bp genome encoding 53 CDSs with no lysogenic, toxin, or tRNA-associated genes. When combined with cefotaxime, the phage effectively inhibited UPEC biofilm formation on disposable rubber catheters. Additionally, phage pretreatment reduced UPEC adhesion to bladder epithelial cells in cellular infection models, demonstrating a dual mechanism of action against both planktonic and adherent bacteria [88].

### Understanding metabolic regulation of tolerance

Choudhary et al. (2024) [89] investigated how glucose concentration (2 mg/mL, consistent with moderate glycosuria) affects UPEC response to gentamicin. They found that glucose reduced gentamicin efficacy and identified mutations in the RNA polymerase sigma factor rpoS following glucose exposure. Gentamicin alone selected for mutations in trkH (potassium ion uptake system) and hyxB (autotransporter). Notably, mutations selected by gentamicin alone were absent when glucose was also present, suggesting that host metabolic conditions fundamentally alter bacterial evolutionary responses to antibiotics. This has implications for treating UTIs in diabetic patients with glycosuria [89].

### Filamentation as a therapeutic target

Peterson et al. (2024) [90] demonstrated that UPEC filamentation during acute UTIs allows bacteria to avoid macrophage engulfment. Using strain UPEC 89, they showed that filamentation occurs independently of the SOS-inducible genes sulA and ymfM. The ability of filaments to evade macrophage engulfment depended on bacterial length, volume, shape, cell surface properties, and environmental factors. Infection-derived filaments were preferentially targeted by macrophages compared to antibiotic-induced filaments, suggesting that the context of filament formation influences immune recognition and potential therapeutic targeting [90].

### Biofilm-targeting approaches

A systematic review by Eskandar (2026) [91] examined emerging therapies targeting UPEC biofilms, including bacteriophage therapy, quorum-sensing inhibitors, and nanoparticle-based drug delivery systems. The review identified that UPEC biofilms enhance antibiotic resistance through extracellular matrix barriers, persister cell formation, efflux pump upregulation, and horizontal gene transfer. Additional approaches such as antibiofilm peptides, probiotics, and immunotherapy also demonstrate potential, though clinical validation remains a critical challenge [91].

### Genomic plasticity as foundation for new strategies

Cortez-De La Puente et al. (2024) [92]reviewed the impact of whole genome sequencing on understanding UPEC pathogenesis, emphasizing that virulence factors are not shared by all UPEC strains, highlighting the importance of personalized approaches to treatment based on genomic characterization [92].

## Clinical translation challenges

Despite recent advances, several critical gaps remain that must be addressed to translate our understanding of phenotypic plasticity into clinical benefit [93].* First,* the specific adhesion phenotypes triggered by different antibiotic classes have not been systematically characterized. Do β-lactams, fluoroquinolones, and aminoglycosides induce distinct morphological changes? Does the clumpy phenotype associated with biofilm-like behavior result preferentially from certain antibiotic classes? These questions remain unanswered yet carry implications for antibiotic selection in patients with recurrent infection history.

*Second*, the functional consequences of antibiotic-induced morphotypes require direct experimental validation. Do bacteria that develop surface roughening following ampicillin exposure exhibit enhanced attachment to bladder epithelial cells? Does filamentation promote intracellular survival? While correlative evidence suggests these relationships exist, mechanistic studies using the 5637-cell model combined with time-lapse microscopy and gentamicin protection assays could establish causal links between antibiotic-induced phenotypes and subsequent pathogenic potential.

*Third*, the extent to which commensal and environmental *E. coli* isolates exhibit similar plastic responses remains largely unexplored. Most studies have focused on established UPEC strains or laboratory strains like *K-12*. However, if commensal isolates from the gut microbiota can undergo similar stress-induced adaptations, this has profound implications for understanding how opportunistic pathogens emerge. A healthy individual carrying commensal *E. coli* exposed to antibiotics for unrelated conditions might harbor bacteria that have acquired enhanced adhesion capabilities, potentially predisposing them to subsequent UTI. Longitudinal studies examining phenotypic plasticity in commensal isolates before and after antibiotic exposure are urgently needed.

*Fourth*, the duration of phenotypic changes following stress removal requires investigation. Are these adaptations rapidly reversible once antibiotic pressure is removed, or do they persist long enough to influence subsequent infection risk? The distinction between transient phenotypic changes and those that become stabilized through epigenetic mechanisms has important implications for understanding recurrence timing and developing therapeutic strategies.

*Fifth*, the interplay between phenotypic plasticity and genetic resistance development remains poorly understood. Do persisted populations formed through phenotypic adaptation serve as reservoirs for the eventual emergence of genetically resistant mutants? The relationship between tolerance (surviving antibiotic exposure without resistance genes) and resistance (genetic changes that reduce susceptibility) may represent sequential stages in bacterial adaptation, with phenotypic plasticity providing a bridge that allows survival until resistance-conferring mutations arise.

## Data Availability

No datasets were generated or analysed during the current study.

## References

[1] Gul S, Durante-Mangoni E. Unraveling the puzzle: health benefits of probiotics—a comprehensive review. J Clinicl Med. 2024;13(5): 1436. 10.3390/jcm13051436. PMC10935031 38592298

[2] Foster-Nyarko E, Pallen MJ. The microbial ecology of *Escherichia coli* in the vertebrate gut. FEMS Microbiol Rev. 2022;46(3):fuac008. 10.1093/femsre/fuac008. PMC9075585 35134909

[3] Chen H. Dual nature of *Escherichia coli*: useful biological tool versus notorious pathogen. Infectious Diseases. 2025. 10.5772/intechopen.1012282.

[4] Martinson JNV, Walk ST. *Escherichia coli* residency in the gut of healthy human adults. EcoSal Plus. 2020;9(1):10.1128/ecosalplus.ESP-0003–2020. 10.1128/ecosalplus.esp-0003-2020. PMC7523338 32978935

[5] Fratamico PM, DebRoy C, Liu Y, Needleman DS, Baranzoni GM, Feng P. Advances in molecular serotyping and subtyping of *Escherichia coli*†. Front Microbiol. 2016. 10.3389/fmicb.2016.00644. PMC4853403 27199968

[6] Clermont O, et al. Characterization and rapid identification of phylogroup G in *Escherichia coli*, a lineage with high virulence and antibiotic resistance potential. Environ Microbiol. 2019;21(8):3107–17. 10.1111/1462-2920.14713. 31188527

[7] Tartof SY, Solberg OD, Manges AR, Riley LW. Analysis of a uropathogenic *Escherichia coli* clonal group by multilocus sequence typing. J Clin Microbiol. 2005;43(12):5860–4. 10.1128/JCM.43.12.5860-5864.2005. PMC1317175 16333067

[8] Beghain J, Bridier-Nahmias A, Le Nagard H, Denamur E, Clermont O. ClermonTyping: an easy-to-use and accurate in silico method for *Escherichia* genus strain phylotyping. Microb Genomics. 2018. 10.1099/mgen.0.000192. PMC6113867 29916797

[9] Wirth T, et al. Sex and virulence in *Escherichia coli*: an evolutionary perspective. Mol Microbiol. 2006;60(5):1136–51. 10.1111/j.1365-2958.2006.05172.x. PMC1557465 16689791

[10] Thakur D, Kumar L. Biofilm-associated *Escherichia coli* infections: pathogenesis, clinical implications, and treatment strategies. Crit Rev Microbiol. 2025;(1):45. 10.1080/1040841X.2025.2548490. 40832759

[11] Gupta K, Mohanty M, Kar P, Nayak G. From gut flora to global threat: the evolutionary story of* Escherichia coli*. Infect Dis. 2025. 10.5772/intechopen.1011897.

[12] Sora VM, Meroni G, Martino PA, Soggiu A, Bonizzi L, Zecconi A. Extraintestinal pathogenic *Escherichia coli*: virulence factors and antibiotic resistance. Pathogens. 2021;10(11): 1355. 10.3390/pathogens10111355. PMC8618662 34832511

[13] Sarowska J, et al. Virulence factors, prevalence and potential transmission of extraintestinal pathogenic* Escherichia coli* isolated from different sources: recent reports. Gut Pathog. 2019;11(1): 10. 10.1186/s13099-019-0290-0. PMC6383261 30828388

[14] Withman B, Gunasekera TS, Beesetty P, Agans R, Paliy O. Transcriptional responses of uropathogenic* Escherichia coli* to increased environmental osmolality caused by salt or urea. Infect Immun. 2013;81(1):80–9. 10.1128/IAI.01049-12. PMC3536127 23090957

[15] De Gaetano GV, Lentini G, Famà A, Coppolino F, Beninati C. In Vivo Role of Two-Component Regulatory Systems in Models of Urinary Tract Infections. Pathogens. 2023;12(1):119. 10.3390/pathogens12010119. PMC9861413 36678467

[16] Kenney LJ, Anand GS. EnvZ/OmpR Two-Component Signaling: An Archetype System That Can Function Noncanonically. EcoSal Plus. 2019;9(1):10.1128/ecosalplus.ESP-0001–2019. 10.1128/ecosalplus.esp-0001-2019. PMC7192543 32003321

[17] Torres-Puig S, et al. ‘Omics’ technologies - what have they told us about uropathogenic *Escherichia coli* fitness and virulence during urinary tract infection? Front Cell Infect Microbiol. 2022;12: 824039. 10.3389/fcimb.2022.824039. PMC8882828 35237532

[18] Rentschler AE, Lovrich SD, Fitton R, Enos-Berlage J, Schwan WR. OmpR regulation of the uropathogenic* Escherichia coli* fimB gene in an acidic/high osmolality environment. Microbiology. 2013;159:316–27. 10.1099/mic.0.059386-0. PMC3709560 23175504

[19] Behzadi P. Classical chaperone-usher (CU) adhesive fimbriome: uropathogenic *Escherichia coli* (UPEC) and urinary tract infections (UTIs). Folia Microbiol (Praha). 2020;65(1):45–65. 10.1007/s12223-019-00719-x. 31165977

[20] Frick-Cheng AE, Shea AE, Roberts JR, Smith SN, Ohi MD, Mobley HLT. Iron limitation induces motility in uropathogenic *E. coli* CFT073 partially through action of LpdA. mBio. 2024;15(7):e01048-24. 10.1128/mbio.01048-24. PMC11253704 38874412

[21] Banerjee R, et al. Tailoring a global iron regulon to a uropathogen. mBio. 2020;11(2):e00351-20. 10.1128/mBio.00351-20. PMC7157518 32209682

[22] Krawczyk B, Wityk P, Burzyńska M, Majchrzak T, Markuszewski MJ. Uropathogenic *Escherichia coli* associated with risk of urosepsis—genetic, proteomic, and metabolomic studies. Int J Mol Sci. 2025;26(12):5681. 10.3390/ijms26125681. PMC12193174 40565144

[23] Thomas L, Abraham T. Disabling iron uptake and pilus assembly in uropathogenic *Escherichia coli* using CRISPR-Cas9: a step towards antivirulence therapy. Antonie Van Leeuwenhoek. 2025;118(8):110. 10.1007/s10482-025-02127-3. 40632173

[24] Wunderle C, et al. Association of leucine and other branched chain amino acids with clinical outcomes in malnourished inpatients: a secondary analysis of the randomized clinical trial EFFORT. Eur J Clin Nutr. 2025;79(1):42–9. 10.1038/s41430-024-01507-8. 39245679

[25] Ahmed J, Ajulo S, Shringi S, Haley BJ, Shah DH. Lactulose-induced transcriptional reprogramming and repression of persistence-associated phenotypes in non-typhoidal *Salmonella enterica*. Pathogens. 2026;15(6):592. 10.3390/pathogens15060592. PMC13304739 42347204

[26] Lapin B, et al. Decoupling shear stress and pressure effects in the biomechanics of autosomal dominant polycystic kidney disease using a perfused kidney-on-chip. Acta Biomater. 2025;197:326–38. 10.1016/j.actbio.2025.03.022. 40089130

[27] Yan C-H, et al. The transcription factor CsgD contributes to engineered *Escherichia coli* resistance by regulating biofilm formation and stress responses. Int J Mol Sci. 2023;24(18):13681. 10.3390/ijms241813681. PMC10530992 37761984

[28] Zhao A, Sun J, Liu Y. Understanding bacterial biofilms: from definition to treatment strategies. Front Cell Infect Microbiol. 2023;13:1137947. 10.3389/fcimb.2023.1137947. PMC10117668 37091673

[29] Geurtsen J, de Been M, Weerdenburg E, Zomer A, McNally A, Poolman J. Genomics and pathotypes of the many faces of *Escherichia coli*. FEMS Microbiol Rev. 2022. 10.1093/femsre/fuac031. PMC9629502 35749579

[30] Mancuso G, Midiri A, Gerace E, Marra M, Zummo S, Biondo C. Urinary tract infections: the current scenario and future prospects. Pathogens. 2023;12(4):623. 10.3390/pathogens12040623. PMC10145414 37111509

[31] Ligon MM, Joshi CS, Fashemi BE, Salazar AM, Mysorekar IU. Effects of aging on urinary tract epithelial homeostasis and immunity. Dev Biol. 2023;493:29–39. 10.1016/j.ydbio.2022.11.003. PMC11463731 36368522

[32] Lee DS, Lee S-J, Choe H-S. Community-acquired urinary tract infection by *Escherichia coli* in the era of antibiotic resistance. BioMed Research International. 2018;2018:1–14. 10.1155/2018/7656752. PMC6178185 30356438

[33] Govindarajan DK, Kandaswamy K. Virulence factors of uropathogens and their role in host pathogen interactions. Cell Surf. 2022;8:100075. 10.1016/j.tcsw.2022.100075. PMC8841375 35198842

[34] Zhang JJ, et al. Paeonol inhibits proliferation and induces cell apoptosis of human T24 and 5637 bladder cancer cells in vitro and in vivo. Clin Transl Oncol. 2021;23(3):601–11. 10.1007/s12094-020-02455-y. 32691366

[35] Andersen TE, Khandige S, Madelung M, Brewer J, Kolmos HJ, Møller-Jensen J. *Escherichia coli* uropathogenesis in vitro: invasion, cellular escape, and secondary infection analyzed in a human bladder cell infection model. Infect Immun. 1858;80(5):1858–67. 10.1128/IAI.06075-11. PMC3347433 22354025

[36] Frömmel U, et al. Adhesion patterns of commensal and pathogenic Escherichia coli from humans and wild animals on human and porcine epithelial cell lines. Gut Pathog. 2013;5(1):31. 10.1186/1757-4749-5-31. PMC4177131 24188314

[37] Andersson DI, Hughes D. Microbiological effects of sublethal levels of antibiotics. Nat Rev Microbiol. 2014;12(7):465–78. 10.1038/nrmicro3270. 24861036

[38] Khan MM, et al. Characterization of clumpy adhesion of *Escherichia coli* to human cells and associated factors influencing antibiotic sensitivity. Microbiol Spectr. 2024;12(5):e02606-23. 10.1128/spectrum.02606-23. PMC11064533 38530058

[39] Martinez JJ. “Type 1 pilus-mediated bacterial invasion of bladder epithelial cells. EMBO J. 2000;19:2803–12. 10.1093/emboj/19.12.2803. PMC203355 10856226

[40] Eto DS, Jones TA, Sundsbak JL, Mulvey MA. Integrin-mediated host cell invasion by type 1–piliated uropathogenic *Escherichia coli*. PLoS Pathog. 2007. 10.1371/journal.ppat.0030100. PMC1914067 17630833

[41] Hogenkamp A, Herías MV, Tooten PCJ, Veldhuizen EJA, Haagsman HP. Effects of surfactant protein D on growth, adhesion and epithelial invasion of intestinal Gram-negative bacteria. Mol Immunol. 2007;44:3517–27. 10.1016/j.molimm.2007.03.013. 17477970

[42] Feenstra T, et al. Adhesion of *Escherichia coli* under flow conditions reveals potential novel effects of fimh mutations. Eur J Clin Microbiol Infect Dis. 2017;36(3):467–78. 10.1007/s10096-016-2820-8. PMC5309269 27816993

[43] Russell SK, et al. Uropathogenic *Escherichia coli* infection-induced epithelial trained immunity impacts urinary tract disease outcome. Nat Microbiol. 2023;8(5):875–88. 10.1038/s41564-023-01346-6. PMC10159856 37037942

[44] Tanaka R, Imai J, Sugiyama E, Tsubaki S, Hozumi K, Tsugawa H. Cyclic-di-AMP confers an invasive phenotype on *Escherichia coli *through elongation of flagellin filaments. Gut Pathog. 2024. 10.1186/s13099-024-00600-4. PMC10809763 38267967

[45] Mukherjee K, Aschenbach W, Hilger A, Saur J, Dobrindt U. Differential perception of virulence factors of uropathogenic Escherichia coli at the level of chromatin dynamics of infected host cells. Front Immunol. 2025;16: 1642683. 10.3389/fimmu.2025.1642683 PMC12535880 41122162

[46] Horvath DJ, et al. Morphological plasticity promotes resistance to phagocyte killing of uropathogenic Escherichia coli. Microbes Infect. 2011;13(5):426–37. 10.1016/j.micinf.2010.12.004. PMC3071881 21182979

[47] Sánchez-Romero MA, Casadesús J. Contribution of phenotypic heterogeneity to adaptive antibiotic resistance. Proc Natl Acad Sci U S A. 2014;111(1):355–60. 10.1073/pnas.1316084111. PMC3890857 24351930

[48] Whelan S, Lucey B, Finn K. Uropathogenic Escherichia coli (UPEC)-Associated Urinary Tract Infections: The Molecular Basis for Challenges to Effective Treatment. Microorganisms. 2023. 10.3390/microorganisms11092169. PMC10537683 37764013

[49] Kalita A, Hu J, Torres AG. Recent advances in adherence and invasion of pathogenic *Escherichia coli*. Curr Opin Infect Dis. 2014;27(5):459–64. 10.1097/QCO.0000000000000092. PMC4169667 25023740

[50] Jarosz D, et al. Stress-driven emergence of heritable non-genetic drug resistance. Nov 28. 2025. 10.21203/rs.3.rs-5744219/v1. *In Review*.

[51] Uzoechi SC, Abu-Lail NI. Changes in cellular elasticities and conformational properties of bacterial surface biopolymers of multidrug-resistant* Escherichia coli* (MDR-E. coli) strains in response to ampicillin. Cell Surf. 2019;5:100019. 10.1016/j.tcsw.2019.100019. PMC6550352 31179402

[52] Uzoechi SC, Abu-Lail NI. Variations in the morphology, mechanics and adhesion of persister and resister *E. coli *Cells in response to ampicillin: AFM Study. Antibiotics (Basel). 2020;9(5): 235. 10.3390/antibiotics9050235. PMC7277365 32392749

[53] Li M, et al. The resistance mechanism of* Escherichia coli *induced by ampicillin in laboratory. Infect Drug Resist. 2019;12:2853–63. 10.2147/IDR.S221212. PMC6750165 31571941

[54] Windels EM, Michiels JE, Fauvart M, Wenseleers T, Van Den Bergh B, Michiels J. Bacterial persistence promotes the evolution of antibiotic resistance by increasing survival and mutation rates. ISME J. 2019;13(5):1239–51. 10.1038/s41396-019-0344-9. PMC6474225 30647458

[55] Pérez-Villalobos V, Vidal R, Hermoso MA, Bustamante P. Persistence phenotype of adherent-invasive *Escherichia coli* in response to ciprofloxacin, revealing high-persistence strains. Microb Cell. 2025;12:173–81. 10.15698/mic2025.07.854. PMC12321266 40761409

[56] Abdelwahed EK, Hussein NA, Moustafa A, Moneib NA, Aziz RK. Gene Networks and Pathways Involved in Escherichia coli Response to Multiple Stressors. Microorganisms. 2022;10(9):1793. 10.3390/microorganisms10091793. PMC9501238 36144394

[57] Salzer A, Wolz C. Role of (p)ppGpp in antibiotic resistance, tolerance, persistence and survival in Firmicutes. microLife. 2023;4:uqad009. 10.1093/femsml/uqad009. PMC10117726 37223729

[58] Bouillet S, Bauer TS, Gottesman S. RpoS and the bacterial general stress response. Microbiol Mol Biol Rev. 2024;88(1):e00151-22. 10.1128/mmbr.00151-22. PMC10966952 38411096

[59] Valencia EY, De Moraes Gomes F, Ospino K, Spira B. RpoS role in antibiotic resistance, tolerance and persistence in *E. coli* natural isolates. BMC Microbiol. 2024;24(1):72. 10.1186/s12866-024-03222-7. PMC11323705 38443813

[60] Potrykus K, Bryszkowska K, Gąsior F, Klasa W. (p)ppGpp: the magic goes on. Microbiol Mol Biol Rev pp. 2026. 10.1128/mmbr.00413-25. e00413-25, Apr. PMC13296368 42053290

[61] Karczewska M, Strzelecki P, Bogucka K, Potrykus K, Szalewska-Pałasz A, Nowicki D. Increased levels of (p)ppGpp correlate with virulence and biofilm formation, but not with growth, in strains of uropathogenic *Escherichia coli*. Int J Mol Sci. 2023;24(4):3315. 10.3390/ijms24043315. PMC9962837 36834725

[62] Lee JH, Zhao Y. ClpXP-dependent RpoS degradation enables full activation of type III secretion system, amylovoran production, and motility in *Erwinia amylovora*. Phytopathology®. 2017;107(11):1346–52. 10.1094/PHYTO-06-17-0198-R. 28691868

[63] Masi M, Pagès J-M. Structure, function and regulation of outer membrane proteins involved in drug transport in Enterobactericeae: the OmpF/C – TolC case. Open Microbiol J. 2013;7(1):22–33. 10.2174/1874285801307010022. PMC3617542 23569467

[64] Masi M, Pinet E, Pagès J-M. Complex response of the CpxAR two-component system to β-lactams on antibiotic resistance and envelope homeostasis in Enterobacteriaceae. Antimicrob Agents Chemother. 2020;64(6):e00291-20. 10.1128/AAC.00291-20. PMC7269474 32229490

[65] Delhaye A, Collet J-F, Laloux G. Fine-tuning of the Cpx envelope stress response is required for cell wall homeostasis in *Escherichia coli*. mBio. 2016. 10.1128/mBio.00047-16. PMC4791840 26908573

[66] Mysorekar IU, Hultgren SJ. Mechanisms of uropathogenic *Escherichia coli* persistence and eradication from the urinary tract. Proceedings of the National Academy of Sciences. 2006;103(38):14170–5. 10.1073/pnas.0602136103. PMC1564066 16968784

[67] Engelhardt F, et al. (p)ppGpp imposes graded transcriptional changes to impair motility and promote antibiotic tolerance in biofilms. NPJ Biofilms Microbiomes. 2025;11(1):148. 10.1038/s41522-025-00795-7. PMC12316884 40750589

[68] Chadha J (2021) In vitro effects of sub-inhibitory concentrations of amoxicillin on physiological responses and virulence determinants in a commensal strain of* Escherichia coli*. J Appl Microbiol. 131(2):682–94. 10.1111/jam.14987. 33387370

[69] Matuła K, et al. Phenotypic plasticity of* Escherichia coli* upon exposure to physical stress induced by ZnO nanorods. Sci Rep. 2019;9(1):8575. 10.1038/s41598-019-44727-w. PMC6561948 31189961

[70] Yang D, et al. Functional Mapping of Phenotypic Plasticity of Staphylococcus aureus Under Vancomycin Pressure. Front Microbiol. 2021;12:696730. 10.3389/fmicb.2021.696730. PMC8458881 34566908

[71] Zhou Y, et al. Urinary tract infections caused by uropathogenic *Escherichia coli*: mechanisms of infection and treatment options. Int J Mol Sci. 2023;24(13): 10537. 10.3390/ijms241310537. PMC10341809 37445714

[72] Karoui ME, Madrid I, Méléard S. Phenotypic plasticity trade-offs in an age-structured model of bacterial growth under stress. arXiv. 2024. 10.48550/ARXIV.2403.13415. PMC13294164 42340457

[73] Lazar V, Oprea E, Ditu L-M. Resistance, Tolerance, Virulence and Bacterial Pathogen Fitness—Current State and Envisioned Solutions for the Near Future. Pathogens. 2023;12(5):746. 10.3390/pathogens12050746. PMC10222143 37242416

[74] Pan Z. Understanding host-microbiome interactions and influence on STEC colonization in cattle using integrated omics. Univ Alta Libr. 2023. 10.7939/R3-JY8Q-4G14.

[75] Maurizi L, et al. Uropathogenic Escherichia coli (UPEC) that hides its identity: features of LC2 and EC73 strains from recurrent urinary tract infections. BMC Microbiol. 2025;25(1):547. 10.1186/s12866-025-04287-8. PMC12376328 40855470

[76] Alshaikh SA, El-banna T, Sonbol F, Farghali MH. Correlation between antimicrobial resistance, biofilm formation, and virulence determinants in uropathogenic *Escherichia coli* from Egyptian hospital. Ann Clin Microbiol Antimicrob. 2024;23(1):20. 10.1186/s12941-024-00679-2. PMC10894499 38402146

[77] Wilczak M, Surman M, Przybyło M. Altered Glycosylation in Progression and Management of Bladder Cancer. Molecules. 2023;28(8):3436. 10.3390/molecules28083436. PMC10146225 37110670

[78] Holíčková A, et al. Response of the urothelial carcinoma cell lines to cisplatin. Int J Mol Sci. 2022;23(20):12488. 10.3390/ijms232012488. PMC9604399 36293346

[79] Hoffmann MJ, et al. The new immortalized uroepithelial cell line HBLAK contains defined genetic aberrations typical of early stage urothelial tumors. Bladder Cancer. 2016;2(4):449–63. 10.3233/BLC-160065. PMC5181672 28035326

[80] Yamin D, et al. Current and future technologies for the detection of antibiotic-resistant bacteria. Diagnostics. 2023;13(20):3246. 10.3390/diagnostics13203246. PMC10606640 37892067

[81] Sintsova A, et al. Genetically diverse uropathogenic *Escherichia coli* adopt a common transcriptional program in patients with UTIs. eLife. 2019;8:e49748. 10.7554/eLife.49748. PMC6802966 31633483

[82] Choi C, Kim DS, Choi JB, Choi T, Lee JW. Mechanisms and clinical implications of bacterial persistence in recurrent urinary tract infections. Investig Clin Urol. 2026;67(2):123. 10.4111/icu.20250656. PMC12956764 41775442

[83] Ipe DS, Horton E, Ulett GC. The basics of bacteriuria: strategies of microbes for persistence in urine. Frontiers in Cellular and Infection Microbiology. 2016. 10.3389/fcimb.2016.00014. PMC4744864 26904513

[84] Pilmis B, et al. Clinical impact of rapid susceptibility testing on Mueller-Hinton Rapid-SIR directly from urine specimens. Eur J Clin Microbiol Infect Dis. 2020;39(7):1373–7. 10.1007/s10096-020-03855-2. 32090300

[85] Berti A, Rose W, Nizet V, Sakoulas G. Antibiotics and innate immunity: a cooperative effort towards the successful treatment of infections. Open Forum Infectious Diseases. 2020. 10.1093/ofid/ofaa302. PMC7423293 32818143

[86] Kobayashi SD, Malachowa N, DeLeo FR. Neutrophils and Bacterial Immune Evasion. J Innate Immun. 2018;10:5–6. 10.1159/000487756. PMC6784029 29642066

[87] Ahmed MN, Porse A, Sommer MOA, Høiby N, Ciofu O. Evolution of antibiotic resistance in biofilm and planktonic *Pseudomonas aeruginosa* populations exposed to subinhibitory levels of ciprofloxacin. Antimicrob Agents Chemother. 2018;62(8):e00320-18. 10.1128/AAC.00320-18. PMC6105853 29760140

[88] Li X, et al. Characterization of a novel phage vB_EcoP_P64441 and its potential role in controlling uropathogenic *Escherichia coli* (UPEC) and biofilms formation. Virology. 2025;609:110570. 10.1016/j.virol.2025.110570. 40367839

[89] Choudhary S, Smith JA, McNally A, Hall RJ (2024) Glucose alters the evolutionary response to gentamicin in uropathogenic *Escherichia coli*. *Microbiology*. 10.1101/2024.11.26.625364. PMC12282228 40153309

[90] Peterson E, et al. The role of bacterial size, shape and surface in macrophage engulfment of uropathogenic *E. coli* cells. PLoS Pathog. 2024;20(9): e1012458. 10.1371/journal.ppat.1012458. PMC11410268 39241059

[91] Eskandar K. The role of uropathogenic *Escherichia coli *biofilms in antibiotic-resistant urinary tract infections: Nanoparticle-based, phage therapy, and quorum-sensing inhibitor approaches. Curr Urol. 2026;20(2):82–8. 10.1097/CU9.0000000000000308. PMC12885708 41668896

[92] Cortez-de La Puente IM, Lozano-Zarain P, Rocha-Gracia RDC, Martínez-de La Peña C, Baca BE, Arenas-Hernández MMDLP. *Escherichia coli* uropatógena: ejemplo de plasticidad genómica. TIP Rev Espec Cienc Quim Biol. 2024. 10.22201/fesz.23958723e.2024.687.

[93] Zhang D, Yin F, Qin Q, Qiao L. Molecular responses during bacterial filamentation reveal inhibition methods of drug-resistant bacteria. Proc Natl Acad Sci U S A. 2023;120(27):e2301170120. 10.1073/pnas.2301170120. PMC10318954 37364094

